# Supplementary material for: Low mutation rate in epaulette sharks is consistent with a slow rate of evolution in sharks
Source: Nat Commun. 2023 Oct 19;14:6628. doi: 10.1038/s41467-023-42238-x (PMC10587355; doi:10.1038/s41467-023-42238-x)
Supplement: Supplementary file 1 — Supplementary Information [file 41467_2023_42238_MOESM1_ESM.pdf]

Supplementary Information

Low mutation rate in epaulette sharks is consistent with a slow rate of evolution in sharks

Ashley T. Sendell-Price[*], Frank J. Tulenko[*], Mats Pettersson, Kang Du, Margo Montandon, Sylke Winkler, Kathleen Kulb, Gavin Naylor, Adam Phillippy, Olivier Federico, Jacquelyn Mountcastle, Jennifer R. Balacco, Amalia Dutra, Rebecca Dale, Bettina Haase, Erich Jarvis, Gene Myers, Shawn M. Burgess, Peter D. Currie, Leif Andersson, Manfred Schartl

[*] Contributed equally

Correspondence to:

phch1@biozentrum.uni-wuerzburg.de; leif.andersson@imbim.uu.se;
peter.currie@monash.edu; burgess@mail.nih.gov

Supplementary Fig. 1. Genome assembly metrics. BlobToolKit snailplot showing N50 metrics and BUSCO gene completeness of the maternal genome assembly. The main plot is divided into 1,000 size-ordered bins around the circumference with each bin representing 0.1% of the assembly. The distribution of scaffold lengths is shown in dark grey with the plot radius scaled to the longest scaffold present in the assembly (shown in red). Orange and pale-orange arcs show the N50 and N90 scaffold lengths, respectively. The pale gray spiral shows the cumulative scaffold count on a log scale with white scale lines showing successive orders of magnitude. The blue and pale-blue area around the outside of the plot shows the distribution of GC, AT and N percentages in the same bins as the inner plot. A summary of complete, fragmented, duplicated and missing BUSCO genes in the vertebrata_odb9 set is shown in the top right.

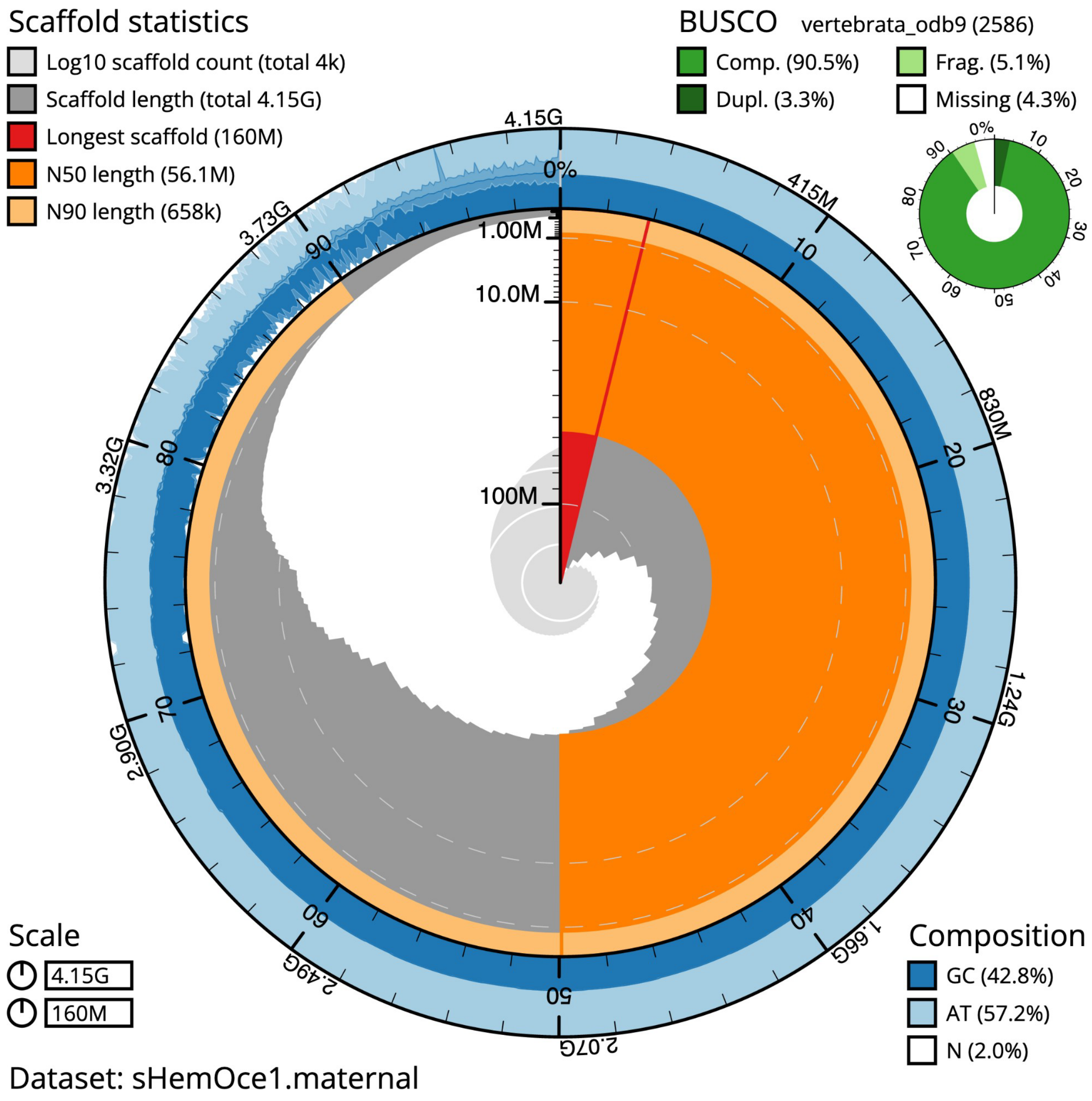

Supplementary Fig. 2. Parental origin of mutation on scaffold_8_mat. The *de novo* mutation identified on scaffold_8_mat (position: 66018494bp) can be determined to be of paternal origin based on the presence of a downstream SNP that is segregating in parental samples. In the focal offspring (ind1983) reads spanning both sites that contain a mutated allele (C) always contain the paternal allele (C) at the segregating site.

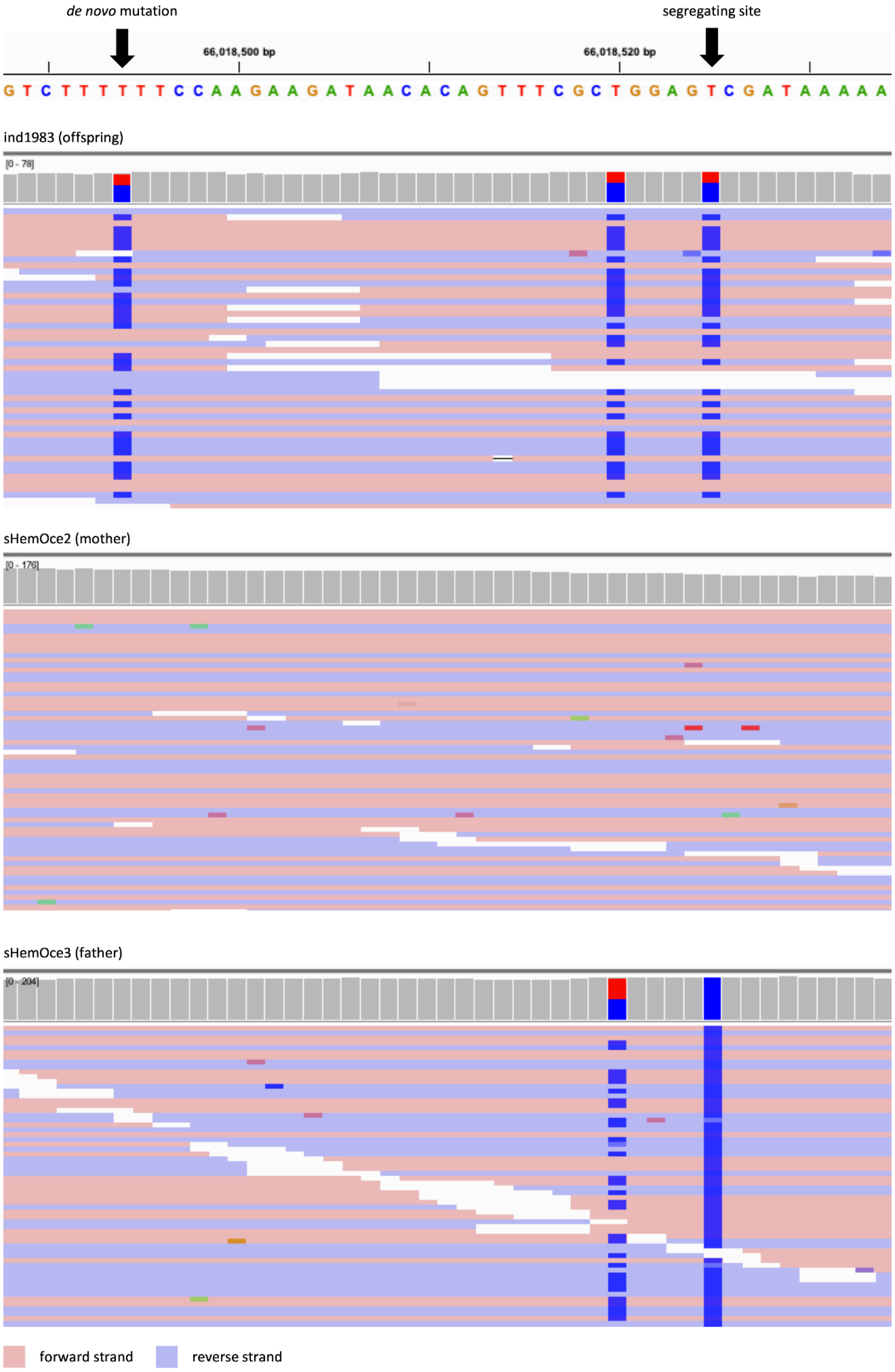

Supplementary Table 1. Directly estimated vertebrate *de novo* mutation rates with PCR confirmation used to generate Fig. 8.

Class	Order	Species	Common Name	Reference	No. trios	Rate ($\mu \times 10e^{-10}$)	95% CI ($10e^{-10}$)
Actinopterygii	Cichliformes	*Astatotilapia calliptera*	Eastern river bream	1	9	35	16 - 46
		Aulonocara stuartgranti	Flavescent peacock	1	9	35	16 - 46
		Lethrinops lethrinus	Scarlet Fin Lethrinus	1	9	35	16 - 46
	Clupeiformes	*Clupea harengus*	Atlantic herring	2	12	20	11 - 29
Aves	Passeriformes	*Ficedula albicollis*	Collared flycatcher	3	7	46	34 - 59
Chondrichthyes	Orectolobiformes	*Hemiscyllium ocellatum*	Epaulette shark	This work	9	7	1.4 - 14.1
Mammalia	Artiodactyla	*Bos taurus*	Cattle	4	5	117	Not reported
	Artiodactyla	*Sus scrofa*	Wild boar	5	5	36	28 - 44
	Carnivora	*Canis lupus*	Wolf	6	4	45	26 - 71
		Felis catus	Domestic cat	7	11	86	75 - 97
	Monotremata	*Ornithorhynchus anatinus*	Platypus	8	2	70	41 - 120
	Primates	*Aotus nancymaae*	Owl monkey	9	14	81	Not reported
		Callithrix jacchus	Marmoset	10	1	43	Not reported
		Chlorocebus sabaeus	Green monkey	11	3	94	Not reported
		Gorilla gorilla	Gorilla	12	2	113	75 - 160
		Homo sapiens	Human	13	1	117	88 - 162
				13	1	97	67 - 134
				14	78	120	Not reported
				15	269	120	Not reported
				16	13	128	113 - 143
				17	719	105	Not reported
				18	1550	129	Not reported
				19	150	128	Not reported
				20	516	130	Not reported

				21	593	110	Not reported
				22	1449	122	108 - 131
		Macaca mulatta	Rhesus macaque	23	14	58	Not reported
				24	19	77	69 - 85
		Microcebus murinus	Grey mouse lemur	25	2	152	128 - 178
		Pan troglodytes	Chimpanzee	26	6	120	Not reported
				27	1	148	Not reported
				12	7	126	95 - 170
		Papio anubis	Baboon	28	12	57	51 - 64
		Pongo abelii	Orangutan	12	1	166	130 - 220
	Rodentia	*Mus musculus*	House mouse	29	8	57	Not reported
				30	15	39	Not reported

Supplementary Table 2. Summary of the pedigree and the sequencing depth used for estimation of the de novo mutation rate. Sequencing depth was estimated using the samtools 'depth' command.

Sample ID	Pedigree	Sequencing depth	Callable 'informative' sites per trio	Accession no.
sHemOce2	Mother	113	-	SAMN36403480
sHemOce3	Father	135.6	-	SAMN36403481
ind1722	Offspring	82	415,534,410	SAMN35791144
ind1835	Offspring	74.7	431,947,407	SAMN35791145
ind1895	Offspring	49.9	426,853,267	SAMN35791146
ind1923	Offspring	95.9	333,716,129	SAMN35791147
ind1925	Offspring	61.9	457,044,582	SAMN35791148
ind1983	Offspring	60.4	426,022,109	SAMN35791149
ind2023	Offspring	70.1	382,847,039	SAMN35791150
ind2024	Offspring	75.9	386,355,463	SAMN35791151
ind2046	Offspring	60.8	431,490,538	SAMN35791152

Supplementary Table 3. Primer pairs used to amplify genomic regions containing candidate *de novo* mutations from the parents and offspring.

**Supplementary table 3a: Primers for offspring**					
**Target candidate mutation**	**Primer set**	**Fragment size**	**Primer name**	**Tm**	**Sequence 5' - 3'**
Scaffold_4_mat: 48132425	PCR primer set	615 bp	1861_HemOce-sc4-3-F	60.07	AAT TCG AAG GAC CGC AGA TGT
			1862_HemOce-sc4-3-R	58.86	AAG GCA GAA CAG AAG TCC CA
	Sequencing primer		1861_HemOce-sc4-3-F	-	AAT TCG AAG GAC CGC AGA TGT
Scaffold_4_mat: 13524629	PCR primer set 1	638 bp	1920_HemOce-sc4-5-F	59.67	CTG CTG CTG CTG AGA TTT ACG
			1921_HemOce-sc4-5-R	59.79	TCT TAT CTG TCA GCC AAG GGC
	Sequencing primer 1		1920_HemOce-sc4-5-F		CTG CTG CTG CTG AGA TTT ACG
Scaffold_8_mat: 66018494	PCR primer set	541 bp	1867_HemOce-sc8-3-F	59.05	GCC TCA ACC ACA ACT TCA GG
			1868_HemOce-sc8-3-R	60.03	GCC CAG CTG TTA TCA ACC CT
	Sequencing primer		1867_HemOce-sc8-3-F	-	GCC TCA ACC ACA ACT TCA GG
Scaffold_14_mat: 4777531	PCR primer set 1	560 bp	1922_HemOce-sc14-1-F	57.71	TGT CCA TGG TGA ACA GCA AT
			1923_HemOce-sc14-1-R	59.38	ACG TAG TTG TGG ACA GAT GCT
	Sequencing primer 1		1923_HemOce-sc14-1-R	-	ACG TAG TTG TGG ACA GAT GCT
	PCR primer set 2	507 bp	1924_HemOce-sc14-2-F	59.45	CAA GTG TGA GCA GCT TGA TTG A
			1925_HemOce-sc14-2-R	59.96	AGT TGT GGA CAG ATG CTC CAT T
	Sequencing primer 2		1925_HemOce-sc14-2-R	-	AGT TGT GGA CAG ATG CTC CAT T
Scaffold_19_mat: 62722441	PCR primer set	603 bp	1873_HemOce-sc19-3-F	60.04	TTC CTG AAG CGT GCT GCA TA
			1874_HemOce-sc19-3-R	60.18	CAT TAC ACA AAC GGT CGC GG
	Sequencing primer		1869_HemOce-sc19-1-F	-	ACT GGA CGC TTC TGC AAT CA
Scaffold_24_mat: 38442575	PCR primer set	558 bp	1877_HemOce-sc24-2-F	59.18	TGG CAA GAT CAC GGA GGT AG
			1878_HemOce-sc24-2-R	59.6	TCT ACA AAC ACT GCC CCA GG
	Sequencing primer		1877_HemOce-sc24-2-F	-	TGG CAA GAT CAC GGA GGT AG
Scaffold_28_mat: 17411576	PCR primer set + Sequencing primer set 1	281 bp	1881_HemOce-sc28-1-F	60.07	ACA GAA TTA CCC CCG GTA GG
			1882_HemOce-sc28-1-R	60.04	CTT TCC AAC CAT CCA GAG GA

	PCR primer set + Sequencing primer set 2	640 bp	1883_HemOce-sc28-2-F	60.18	TAC CCC CGG TAG GGA AAC AA
			1884_HemOce-sc28-2-R	59.97	CAG CTC GGG TGT AGT TTG GT
	PCR primer set + Sequencing primer set 3	536 bp	1885_HemOce-sc28-3-F	59.89	AGA GGA GGG CAA AAA GGT GG
			1886_HemOce-sc28-3-R	60.11	TAG GCC ATG GGT ACC TCT CC
Scaffold_36_mat: 8326154	PCR primer set	576 bp	1889_HemOce-sc36-2-F	59.5	AAA GCA GAA AAC CTT GTG CAG T
			1890_HemOce-sc36-2-R	60.39	TGG GCC ACG ATC AAT GTC TG
	Sequencing primer		1889_HemOce-sc36-2-F	-	AAA GCA GAA AAC CTT GTG CAG T
Scaffiold_51_mat: 8719242	PCR primer set	642 bp	1926_HemOce-sc51-1-F	60.88	CTC GGC GAA CAG AAC CAC AA
			1927_HemOce-sc51-1-R	59.08	TGG TCC TCT ATC TGT CGG GA
	Sequencing primer		1927_HemOce-sc51-1-R	-	TGG TCC TCT ATC TGT CGG GA
Scaffold_158_mat: 387249	PCR primer set	734 bp	1930_HemOce-sc158-1-F	59.75	GGT CAG GGA AGC TGA ACG AT
			1931_HemOce-sc158-1-R	60.18	TGA TGA AGG GCT TTT GCC CA
	Sequencing primer		1930_HemOce-sc158-1-F	-	GGT CAG GGA AGC TGA ACG AT

Supplementary table 3b: Primers for parents

Target candidate mutation	Primer set	Fragment size	Primer name	Tm	Sequence 5' - 3'
Scaffold_4_mat: 48132425	Primer set 1	278 bp	1857_HemOce-sc4-1-F	59.96	GCA CCA CCA CCT CCA TTA CA
			1858_HemOce-sc4-1-R	57.9	TGG TCC AGT CAT GTT TGT TGT
	Primer set 2	578 bp	1859_HemOce-sc4-2-F	60.11	TTC GAA GGA CCG CAG ATG TC
			1860_HemOce-sc4-2-R	60.03	CCT CCT GCA GGG GCA TTA AA
	Primer set 3	615 bp	1861_HemOce-sc4-3-F	60.07	AAT TCG AAG GAC CGC AGA TGT
			1862_HemOce-sc4-3-R	58.86	AAG GCA GAA CAG AAG TCC CA
Scaffold_4_mat: 13524629	Primer set 1	544 bp	1918_HemOce-sc4-4-F	60.14	GGT GAA AAC GTG TTG CTG GTT
			1919_HemOce-sc4-4-R	59.38	TTC CCA CAC TGA TCC AGC TC
	Primer set 2	638 bp	1920_HemOce-sc4-5-F	59.67	CTG CTG CTG CTG AGA TTT ACG
			1921_HemOce-sc4-5-R	59.79	TCT TAT CTG TCA GCC AAG GGC

	Primer set 3	681 bp	1920_HemOce-sc4-5-F	59.67	CTG CTG CTG CTG AGA TTT ACG
			1920A_HemOce-sc4-6-R	59	CAG AGG CTG CTC TTG GAG TG
Scaffold_8_mat: 66018494	Primer set 1	329 bp	1863_HemOce-sc8-1-F	60.67	GGC ACG ACC ACA GCA TCT T
			1864_HemOce-sc8-1-R	60.18	CGC ATT TTA GCC TGT GGC TTT AT
	Primer set 2	532 bp	1865_HemOce-sc8-2-F	59.68	TAC TTC AGA CAC CAG GCA CG
			1866_HemOce-sc8-2-R	59.68	GCC TTC TGT CTC TTG CCT CA
	Primer set 3	541 bp	1867_HemOce-sc8-3-F	59.05	GCC TCA ACC ACA ACT TCA GG
			1868_HemOce-sc8-3-R	60.03	GCC CAG CTG TTA TCA ACC CT
Scaffold_14_mat: 4777531	Primer set 1	560 bp	1922_HemOce-sc14-1-F	57.71	TGT CCA TGG TGA ACA GCA AT
			1923_HemOce-sc14-1-R	59.38	ACG TAG TTG TGG ACA GAT GCT
	Primer set 2	507 bp	1924_HemOce-sc14-2-F	59.45	CAA GTG TGA GCA GCT TGA TTG A
			1925_HemOce-sc14-2-R	59.96	AGT TGT GGA CAG ATG CTC CAT T
	Primer set 3	596 bp	1924_HemOce-sc14-2-F	59.45	CAA GTG TGA GCA GCT TGA TTG A
			1924A_HemOce-sc 14-3-R	58	GAA AGA TGC TTT GAT TTA CAT GCA CC
Scaffold_19_mat: 62722441	Primer set 1	279 bp	1869_HemOce-sc19-1-F	59.96	ACT GGA CGC TTC TGC AAT CA
			1870_HemOce-sc19-1-R	59.32	GCT GAC CAC AAA AGA AGC AGT
	Primer set 2	748 bp	1871_HemOce-sc19-2-F	60.03	TGC ATG CCA CGG TCC TAT TT
			1872_HemOce-sc19-2-R	59.96	TCC ATT GAA CAC AGC CCT CC
	Primer set 3	603 bp	1873_HemOce-sc19-3-F	60.04	TTC CTG AAG CGT GCT GCA TA
			1874_HemOce-sc19-3-R	60.18	CAT TAC ACA AAC GGT CGC GG
Scaffold_24_mat: 38442575	Primer set 1	303 bp	1875_HemOce-sc24-1-F	60.09	TCT GGC TGT TCC TTT TTG AGG T
			1876_HemOce-sc24-1-R	59.96	CTT CGC CCA ACA GTT CCT CT
	Primer set 2	558 bp	1877_HemOce-sc24-2-F	59.18	TGG CAA GAT CAC GGA GGT AG
			1878_HemOce-sc24-2-R	59.6	TCT ACA AAC ACT GCC CCA GG
	Primer set 3	545 bp	1879_HemOce-sc24-3-F	59.39	CTG GTC ACA GAC CTC CAG TC
			1880_HemOce-sc24-3-R	60.25	ACT TCG CCC AAC AGT TCC TC
Scaffold_28_mat: 17411576	Primer set 1	281 bp	1881_HemOce-sc28-1-F	60.07	ACA GAA TTA CCC CCG GTA GG

			1882_HemOce-sc28-1-R	60.04	CTT TCC AAC CAT CCA GAG GA
	Primer set 2	640 bp	1883_HemOce-sc28-2-F	60.18	TAC CCC CGG TAG GGA AAC AA
			1884_HemOce-sc28-2-R	59.97	CAG CTC GGG TGT AGT TTG GT
	Primer set 3	536 bp	1885_HemOce-sc28-3-F	59.89	AGA GGA GGG CAA AAA GGT GG
			1886_HemOce-sc28-3-R	60.11	TAG GCC ATG GGT ACC TCT CC
Scaffold_36_mat: 8326154	Primer set 1	331 bp	1887_HemOce-sc36-1-F	57.94	GCA AAA GTA TGA GTT GAA TGC CT
			1888_HemOce-sc36-1-R	58.6	TTC CCT CCT CTT CCT CTC CT
	Primer set 2	576 bp	1889_HemOce-sc36-2-F	59.5	AAA GCA GAA AAC CTT GTG CAG T
			1890_HemOce-sc36-2-R	60.39	TGG GCC ACG ATC AAT GTC TG
	Primer set 3	677 bp	1891_HemOce-sc36-3-F	58.23	AGC AGA AAA CCT TGT GCA GT
			1892_HemOce-sc36-3-R	59.76	GCT CAC TAG CGT GAG GAA CA
Scaffiold_51_mat: 8719242	Primer set 1	642 bp	1926_HemOce-sc51-1-F	60.88	CTC GGC GAA CAG AAC CAC AA
			1927_HemOce-sc51-1-R	59.08	TGG TCC TCT ATC TGT CGG GA
	Primer set 2	626 bp	1928_HemOce-sc51-2-F	60.33	ACA GCC CTC CAA TCT CCG TA
			1929_HemOce-sc51-2-R	59.96	AAA GGG AGC ATT CAG CGT CA
Scaffold_158_mat: 387249	Primer set 1	734 bp	1930_HemOce-sc158-1-F	59.75	GGT CAG GGA AGC TGA ACG AT
			1931_HemOce-sc158-1-R	60.18	TGA TGA AGG GCT TTT GCC CA
	Primer set 2	608 bp	1932_HemOce-sc158-2-F	58.92	TTG GTA AAG CAT TTC TGT CTC CC
			1933_HemOce-sc158-2-R	60.6	GAC CTG CTT TGC TTT TCC AGC

SUPPLEMENTARY REFERENCES

1. Malinsky, M. et al. Whole-genome sequences of Malawi cichlids reveal multiple radiations interconnected by gene flow. *Nature Ecology & Evolution* 2, 1940–1955 (2018).

2. Feng, C. et al. Moderate nucleotide diversity in the Atlantic herring is associated with a low mutation rate. *Elife* 6, e23907 (2017).

3. Smeds, L., Qvarnström, A. & Ellegren, H. Direct estimate of the rate of germline mutation in a bird. *Genome Res*. 26, 1211–1218 (2016).

4. Harland, C. et al. Frequency of mosaicism points towards mutation-prone early cleavage cell divisions in cattle. *bioRxiv* 079863 (2017).

5. Zhang, M., Yang, Q., Ai, H. & Huang, L. Revisiting the evolutionary history of pigs via De Novo mutation rate estimation in a three-generation pedigree. *Genomics Proteomics Bioinformatics* (2022) doi:10.1016/j.gpb.2022.02.001.

6. Koch, E. M. et al. De Novo Mutation Rate Estimation in Wolves of Known Pedigree. *Mol. Biol. Evol*. 36, 2536–2547 (2019).

7. Wang, R. J. et al. De novo mutations in domestic cat are consistent with an effect of reproductive longevity on both the rate and spectrum of mutations. *bioRxiv* 2021.04.06.438608 (2021).

8. Martin, H. C. et al. Insights into Platypus Population Structure and History from Whole-Genome Sequencing. *Mol. Biol. Evol*. 35, 1238–1252 (2018).

9. Thomas, G. W. C. et al. Reproductive Longevity Predicts Mutation Rates in Primates. *Curr. Biol*. 28, 3193–3197.e5 (2018).

10. Yang, C. et al. Evolutionary and biomedical insights from a marmoset diploid genome assembly. *Nature* 594, 227–233 (2021).

11. Pfeifer, S. P. Direct estimate of the spontaneous germ line mutation rate in African green monkeys. *Evolution* 71, 2858–2870 (2017).

12. Besenbacher, S., Hvilsom, C., Marques-Bonet, T., Mailund, T. & Schierup, M. H. Direct estimation of mutations in great apes reconciles phylogenetic dating. *Nat. Ecol. Evol* 3, 286–292 (2019).

13. Conrad, D. F. et al. Variation in genome-wide mutation rates within and between human families. *Nat. Genet*. 43, 712–714 (2011).

14. Kong, A. et al. Rate of de novo mutations, father‘s age, and disease risk. *Nature* 488, 471–475 (2012).

15. Francioli, L. C. et al. Genome-wide patterns and properties of de novo mutations in humans. *Nat. Genet*. 47, 822–826 (2015).

16. Rahbari, R. et al. Timing, rates and spectra of human germline mutation. *Nat. Genet*. 48, 126–133 (2016).

17. Wong, W. S. W. et al. New observations on maternal age effect on germline de novo mutations. *Nat. Commun*. 7, 10486 (2016).

18. Jónsson, H. et al. Parental influence on human germline de novo mutations in 1,548 trios from Iceland. *Nature* 549, 519–522 (2017).

19. Maretty, L. et al. Sequencing and de novo assembly of 150 genomes from Denmark as a population reference. *Nature* 548, 87–91 (2017).

20. Turner, T. N. et al. Genomic Patterns of De Novo Mutation in Simplex Autism. *Cell* 171, 710–722.e12 (2017).

21. Sasani, T. A. et al. Large, three-generation human families reveal post-zygotic mosaicism and variability in germline mutation accumulation. *Elife* 8, e46922 (2019).

22. Kessler, M. D. et al. De novo mutations across 1,465 diverse genomes reveal mutational insights and reductions in the Amish founder population. *Proc. Natl. Acad. Sci. U. S. A*. 117, 2560–2569 (2020).

23. Wang, R. J. et al. Paternal age in rhesus macaques is positively associated with germline mutation accumulation but not with measures of offspring sociability. *Genome Res*. 30, 826–834 (2020).

24. Bergeron, L. A. et al. The germline mutational process in rhesus macaque and its implications for phylogenetic dating. *Gigascience* 10, giab029 (2021).

25. Campbell, C. R. et al. Pedigree-based and phylogenetic methods support surprising patterns of mutation rate and spectrum in the gray mouse lemur. *Heredity* 127, 233–244 (2021).

26. Venn, O. et al. Nonhuman genetics. Strong male bias drives germline mutation in chimpanzees. *Science* 344, 1272–1275 (2014).

27. Tatsumoto, S. et al. Direct estimation of de novo mutation rates in a chimpanzee parent-offspring trio by ultra-deep whole genome sequencing. *Sci. Rep*. 7, 13561 (2017).

28. Wu, F. L. et al. A comparison of humans and baboons suggests germline mutation rates do not track cell divisions. *PLoS Biol.* 18, e3000838 (2020).

29. Milholland, B. et al. Differences between germline and somatic mutation rates in humans and mice. *Nat. Commun*. 8, 15183 (2017).

30. Lindsay, S. J., Rahbari, R., Kaplanis, J., Keane, T. & Hurles, M. E. Similarities and differences in patterns of germline mutation between mice and humans. *Nat. Commun*. 10, 4053 (2019).
