# Supplementary material for: Low mutation rate in epaulette sharks is consistent with a slow rate of evolution in sharks
Source: Nat Commun. 2023 Oct 19;14:6628. doi: 10.1038/s41467-023-42238-x (PMC10587355; doi:10.1038/s41467-023-42238-x)
Supplement: Supplementary file 3 — Reporting Summary [file 41467_2023_42238_MOESM3_ESM.pdf]

nature portfolio

Corresponding author(s): Shawn M. Burgess (orcid: 0000-0003-1147-0596), Peter D. Currie (orcid: 0000-0001-8874-8862), Leif Andersson (orcid: 0000-0002-4085-6968), Manfred Schartl (orcid: 0000-0001-9882-5948)

Last updated by author(s): 25.08.2023

Reporting Summary

Nature Portfolio wishes to improve the reproducibility of the work that we publish. This form provides structure for consistency and transparency in reporting. For further information on Nature Portfolio policies, see our Editorial Policies and the Editorial Policy Checklist.

Statistics

For all statistical analyses, confirm that the following items are present in the figure legend, table legend, main text, or Methods section.

n/a	Confirmed	
☐	☒	The exact sample size (*n*) for each experimental group/condition, given as a discrete number and unit of measurement
☒	☐	A statement on whether measurements were taken from distinct samples or whether the same sample was measured repeatedly
☐	☒	The statistical test(s) used AND whether they are one- or two-sided<br>*Only common tests should be described solely by name; describe more complex techniques in the Methods section.*
☒	☐	A description of all covariates tested
☐	☒	A description of any assumptions or corrections, such as tests of normality and adjustment for multiple comparisons
☐	☒	A full description of the statistical parameters including central tendency (e.g. means) or other basic estimates (e.g. regression coefficient) AND variation (e.g. standard deviation) or associated estimates of uncertainty (e.g. confidence intervals)
☒	☐	For null hypothesis testing, the test statistic (e.g. *F*, *t*, *r*) with confidence intervals, effect sizes, degrees of freedom and *P* value noted<br>*Give P values as exact values whenever suitable.*
☒	☐	For Bayesian analysis, information on the choice of priors and Markov chain Monte Carlo settings
☒	☐	For hierarchical and complex designs, identification of the appropriate level for tests and full reporting of outcomes
☒	☐	Estimates of effect sizes (e.g. Cohen's *d*, Pearson's *r*), indicating how they were calculated

Our web collection on statistics for biologists contains articles on many of the points above.

Software and code

Policy information about availability of computer code

Data collection	no software was used for data collection
Data analysis	The following software was used for data analysis: TrioCanu v. 2.1; purge_dups v.1.2.5; scaff10x v.4.2; GATK; SomatoSim; Bionano Solve v.3.6.1_11162020; Salsa2 HiC v. 2.2; minimap2; variantCaller v.2.3.3; Longranger v.2.2.2; freebayes v.1.3; Merfin; Merqury; mitoVGP; WindowMasker; Exonerate; Genewise; HISAT; StringTie; Trinity; Splign; AUGUSTUS; blast; BWA-mem2; Repeat Masker v4.1.0; GENMAP; samtools; bcftools; picardtools; Geneious Prime. Code used for the detection of candidate de novo mutations and estimation of mutation rate are available at: https://github.com/LeifAnderssonLab/Epaulette_shark_mutation_rate.

For manuscripts utilizing custom algorithms or software that are central to the research but not yet described in published literature, software must be made available to editors and reviewers. We strongly encourage code deposition in a community repository (e.g. GitHub). See the Nature Portfolio guidelines for submitting code & software for further information.

Data

Policy information about availability of data

All manuscripts must include a data availability statement. This statement should provide the following information, where applicable:

- Accession codes, unique identifiers, or web links for publicly available datasets
- A description of any restrictions on data availability
- For clinical datasets or third party data, please ensure that the statement adheres to our policy

Data used for genome assembly construction are available at: https://genomeark.github.io/genomeark-all/Hemiscyllium_ocellatum.html. Additional raw Illumina sequencing reads used for detection of candidate de novo mutations have been deposited at NCBI under the BioProject PRJNA900175 (https://www.ncbi.nlm.nih.gov/bioproject/PRJNA900175). Genome assemblies (paternal and maternal haplotypes) are available from NCBI under GenBank accession numbers GCA_020745735.1 (https://www.ncbi.nlm.nih.gov/assembly/GCA_020745735.1) and GCA_020745765.1 (https://www.ncbi.nlm.nih.gov/assembly/GCA_020745765.1). Both haplotypes are also available through the UCSC genome browser gateway (https://genome.ucsc.edu/h/GCA_020745735.1 and https://genome.ucsc.edu/h/GCA_020745765.1).

Research involving human participants, their data, or biological material

Policy information about studies with human participants or human data. See also policy information about sex, gender (identity/presentation), and sexual orientation and race, ethnicity and racism.

Reporting on sex and gender	not applicable
Reporting on race, ethnicity, or other socially relevant groupings	not applicable
Population characteristics	not applicable
Recruitment	not applicable
Ethics oversight	not applicable

Note that full information on the approval of the study protocol must also be provided in the manuscript.

Field-specific reporting

Please select the one below that is the best fit for your research. If you are not sure, read the appropriate sections before making your selection.

☐ Life sciences ☐ Behavioural & social sciences ☒ Ecological, evolutionary & environmental sciences

For a reference copy of the document with all sections, see nature.com/documents/nr-reporting-summary-flat.pdf

Ecological, evolutionary & environmental sciences study design

All studies must disclose on these points even when the disclosure is negative.

Study description	To generate a reference genome of Epaulette shark, and determine a pedigree-based estimate of the de novo mutation rate. Mutation rate was calculated based on whole genome sequencing of one breeding pair (mother and father) and nine offspring.
Research sample	Epaulette sharks (Hemiscyllium ocellatum) were selected as a small sized shark amendable to captive breeding. Wild-caught adult epaulette sharks were sourced from the commercial supplier Cairns Marine (Queensland Australia). A single adult male and female breeding pair was isolated in the Monash University epaulette breeding colony for the purpose of collection of eggs of known parentage for pedigree analysis. Both adults were reproductively mature but because they were wild caught were of unknown age. Four male and five female offspring were used to calculate the de novo mutation rate. The age range of the offspring were pre-hatching stages 37 to 38.
Sampling strategy	The isolated breeding pair of adult Epaulette sharks was maintained in a closed, recirculating marine system in a single 2100L tank housed indoors in the Monash University Aquacore facility. Blood samples were collected from the adult ventral tail vein using a 21G needle. For drawing blood, adults were briefly sedated with Aqui-S at a concentration of 30ppm in sea water. Blood was frozen on dry ice, stored at -80C, and used for subsequent DNA isolation. Eggs were collected from the isolated breeding pair, tagged with their date of deposition and origin, and transferred to a separate marine aquarium on the same sea water system. Eggs were reared to pre-hatching stages. Offspring were then snap frozen, stored at -80C, and used for subsequent DNA isolation. No quantitative data were generated. Mutations were confirmed by Sanger sequencing, thus no further individuals needed to be analyzed.
Data collection	Adult blood was collected by F.T. in collaboration with an aquarium veterinarian. Epaulette eggs from the breeding pair were tagged with tape and marker and transferred to a separate rearing aquaria by Monash aquacore staff. Pre-hatching stage offspring were subsequently flash frozen by F.T. and R.D. and stored at -80C. Data recorded by pen and paper.
Timing and spatial scale	Epaulette adult male and female parents were paired together beginning 21 November 2017 and remained isolated together for the

	duration of egg collection. Eggs were collected over a continuous period from October 2018 to September 2019 and reared for pre-hatching stage offspring, which were used for DNA isolation. Timing and collection dates was dependent on laying dates for the offspring.
Data exclusions	No data were excluded
Reproducibility	The trio genome strategy provided reproducibility and accuracy of the genome resource. 9 offspring were resequenced for the de novo mutation rate estimation. No quantitative data were generated in this study.
Randomization	Randomization was not applicable as we targeted analysis of eggs of known parentage.
Blinding	Blinding was not applicable as we targeted analysis of eggs of known parentage.

Did the study involve field work? ☐ Yes ☒ No

Reporting for specific materials, systems and methods

We require information from authors about some types of materials, experimental systems and methods used in many studies. Here, indicate whether each material, system or method listed is relevant to your study. If you are not sure if a list item applies to your research, read the appropriate section before selecting a response.

Materials & experimental systems

n/a	Involved in the study
☒	☐ Antibodies
☒	☐ Eukaryotic cell lines
☒	☐ Palaeontology and archaeology
☐	☒ Animals and other organisms
☒	☐ Clinical data
☒	☐ Dual use research of concern
☒	☐ Plants

Methods

n/a	Involved in the study
☒	☐ ChIP-seq
☒	☐ Flow cytometry
☒	☐ MRI-based neuroimaging

Animals and other research organisms

Policy information about studies involving animals; ARRIVE guidelines recommended for reporting animal research, and Sex and Gender in Research

Laboratory animals	No laboratory animals were used in the study.
Wild animals	Wild-caught adult epaulette sharks (Hemiscyllium ocellatum) were sourced from the supplier Cairns Marine (Queensland Australia) and maintained in an Epaulette breeding colony at Monash University, Clayton Campus. The adults were reproductively mature when brought into the breeding colony but their specific ages are unknown. Following completion of egg collection for this work, the adult breeding pair remained as part of the Monash University Epaulette breeding colony.
Reporting on sex	The nine prehatching stage epaulette shark offspring analyzed included four male and five females.
Field-collected samples	The Epaulette shark mating pair was maintained in a 2100L tank as part of closed, recirculating marine system housed indoors in the Monash University, Clayton Campus, Aquacore facility. Water temperature was maintained at approximately 25C and a graded light cycle was used to mimic sunrise and sunset with a 12-hour photoperiod. Adult sharks were fed a mixed diet that included glassies, pilchard, whiting, pipis and squid four times per week. Laid eggs were tagged and transferred to a separate glass aquaria on the same marine sea water system as the adults, and reared to pre-hatching stages for tissue collection and DNA isolation. Following the end of egg collection, the adult breeding pair remained as part of the Monash University Epaulette breeding colony.
Ethics oversight	Epaulette husbandry, breeding, and egg collection were carried out in accordance with approved Monash University Animal Ethics Project ID 30347, and blood samples from adult sharks were collected according to approved Monash University Animal Ethics Project ID 13945.

Note that full information on the approval of the study protocol must also be provided in the manuscript.
